# Knowledge of Community General Practitioners and Nurses on Pre-Hospital Stroke Prevention and Treatment in Chongqing, China

**DOI:** 10.1371/journal.pone.0138476

**Published:** 2015-09-18

**Authors:** Juan Yang, Jie Zhang, Shu Ou, Ni Wang, Jian Wang

**Affiliations:** 1 Department of Neurology, the Second People’s Hospital of Chengdu, Chengdu, Sichuan, China; 2 Department of Neurology, Chongqing Emergency Medical Center, Chongqing, China; 3 Department of Neurology, the Second Affiliated Hospital of Chongqing Medical University, Chongqing, China; UNIFESP Federal University of São Paulo, BRAZIL

## Abstract

**Background and Purpose:**

This study aimed to investigate the knowledge of community general practitioners (GPs) and nurses about pre-hospital stroke recognition, treatment and management and secondary stroke prevention; to identify the sociodemographic and educational factors influencing knowledge.

**Methods:**

A self-designed test questionnaire was applied in a self-administered close-exam setting among 480 GPs and nurses working in community health centers (stations) in eight urban districts of Chongqing.

**Results:**

A total of 331 (69%) valid test questionnaires were returned. Of the 331 participants, 39% were aware of the clinical guidelines for cerebrovascular diseases, whereas 48% considered themselves to have stroke management capabilities. The correct rate of answering questions of pre-hospital recognition and management knowledge was as low as 24%, the correct rate of secondary stroke prevention knowledge was only 38%. In terms of the total score for stroke prevention and treatment knowledge, there were significant differences between the medical staff with different specialties before engaging in community health services and whether they have received GP training (*P* <0.05).

**Conclusion:**

The community GPs and nurses in the urban districts of Chongqing clearly lack knowledge of stroke, and the levels of stroke prevention and treatment urgently need to be improved.

## Introduction

To date, cerebrovascular diseases have become the third leading cause of death and the leading cause of adult disability in the world [1]. However, in China stroke is the leading cause of death in the national population [2]. Numerous studies have shown that improving modifiable risk factors for stroke, including hypertension, diabetes, hyperlipidemia, and smoking, can reduce the incidence of the disease [3–6]. Active intervention for stroke patients can effectively reduce the recurrence and mortality of the disease [7]. Early stroke treatment is key to reducing mortality and improve the outcomes, especially for ischemic stroke, because in patients eligible for intravenous fibrinolytic therapy, benefit of therapy is time dependent, and treatment should be initiated as quickly as possible [8]. National and international clinical guidelines for diagnosis and treatment of acute ischemic stroke all emphasize pre-hospital management of stroke patients, including pre-hospital stroke recognition, on-site treatment, and transfer [8–9]. In the existing model of community health service in China, community general practitioners (GPs) and nurses play important roles in pre-hospital stroke recognition and management, as well as secondary stroke prevention [10]. This study aimed to investigate the knowledge of community GPs and nurses about pre-hospital stroke recognition, treatment and management and secondary stroke prevention; to identify the sociodemographic and educational factors influencing knowledge.

## Materials and Methods

### Setting and Sampling

This is a cross-sectional study. 480 GPs and nurses from 84 community health centers and 251 community health stations in eight urban districts of Chongqing were randomly selected between June to December 2011. The urban districts included Yuzhong, Jiangbei, Dadukou, Shapingba, Jiulongpo, Nan’an, Yubei and Banan District.

A clustered sampling method was conducted to randomly select 60 of the 335 community health centers (stations) mentioned above, then a random-number grid was used to select eight eligible GPs and nurses in each community health centers (stations) according to the GPs and nurses’ job number. Prior to the survey, all participants were informed that it was voluntary to participate this survey. In addition, all participants were informed of the purpose and significance of the study. Each participant who agreed to participate signed an informed consent. The questionnaire was filled in by way of a closed-exam setting. The participants were not allowed to look up the answers, additionally, the whole process was supervised in the scene by the uniformly trained investigators. All the data were anonymized. This study was approved by the ethics committees of the second affiliated hospital, Chongqing Medical University.

### Data collection instrument

A self-designed test questionnaire was developed in accordance with the 2010 Chinese Clinical Guideline for Diagnosis and Treatment of Acute Ischemic Stroke [9] and the 2010 Chinese Guideline for Secondary Prevention of Ischemic Stroke and Transient Ischemic Attack (TIA) [11]. The test questionnaire included general information on the participants (gender, age, occupation, educational level, job titles, specialty before engaging in community health services, GP training, clinical experience time, time since engaging in community health services, workplace, and perceived ability to manage stroke patients in community) and tested the participants’ knowledge about pre-hospital stroke recognition and the management of secondary stroke prevention. Score points were designed as follows: Four questions were included for pre-hospital stroke recognition and management knowledge, involving a total score of 14 points. (1) Knowledge of stroke warning signs (5 points): The participant should correctly answer five sudden signs of stroke, including “sudden slurred speech, speaking difficulties, or understanding difficulties; sudden blurred vision of one or both eyes; sudden numbness or weakness in one side of the face or limbs; sudden severe headache without known cause; and sudden walking difficulties, dizziness, balance disorders or incoordination”. Each scored 1 point. (2) Knowing the use of pre-hospital stroke assessment methods (1 point): A correct answer using the methods of “Cincinnati pre-hospital Stroke Screen or Los Angeles pre-hospital Stroke Screen” scored 1 point. (3) Awareness of thrombolytic therapy and its time window (2 points): The participant was asked “What is the most effective treatment for acute ischemic stroke?” and “What is the effective time window of this treatment?” The correct answer was “thrombolysis or rt-PA” and "3–4.5 h” respectively. (4) Pre-hospital management of acute stroke patients (6 points): The participant should correctly answer six measures of pre-hospital emergency management, including “management of airway, breathing and circulation problems; monitoring of the heart; establishment of intravenous access; inhaled oxygen supply; assessment of the presence or absence of hypoglycemia; and transfer of patient to a nearby qualified hospital as soon as possible”. Seven questions were included for secondary stroke prevention knowledge, providing a total score of 22 points. (1) Modifiable major risk factors for stroke (5 points): The scoring criteria were that the participant should be able to state the five major risk factors for stroke, such as hypertension, diabetes, dyslipidemia, heart disease and unhealthy lifestyle (including smoking and lack of physical activity). (2) Definition and intervention of transient ischemic attack (TIA) (2 points): TIA is defined as “a transient episode of neurological dysfunction caused by ischemia–focal brain, spinal cord, or retinal–without acute infarction”. Once TIA is recognized in a community hospital, the correct practice is to “transfer patient to a qualified hospital for further examination as soon as possible to prevent the onset of complete stroke”. (3) Blood pressure (BP) control for secondary prevention of ischemic stroke or TIA (3 points): The goal is generally to reduce BP to “≤140/90 mmHg” and ideally ≤130/80 mmHg. For hypertensive patients with diabetes, the goal is to lower BP to “≤130/80 mmHg” (4) Blood sugar control (1 point): The target level of blood sugar control for diabetes is “hemoglobin A1C (HbA1C) <6.5%”. Low blood glucose should be avoided in high-risk patients with type 2 diabetes. (5) Blood lipid control (5 points): Ischemic stroke and TIA patients with elevated cholesterol levels are recommended for the use of “statins”, and the goal is to reduce low-density-lipoprotein cholesterol (LDL-C) “to <2.59 mmol/L or by 30–40%”; the LDL-C level should be reduced to “<2.07 mmol/L or by >40%” among patients associated with multiple risk factors or evidence for intracranial and extracranial aortic atherosclerotic vulnerable plaque or arterial embolism. The main side effects of statins include “abnormal liver function” and “muscle damage”. (6) Prevention of atrial fibrillation-related cerebral embolism (2 points): For ischemic stroke and TIA patients associated with atrial fibrillation, the use of “warfarin” is recommended to prevent stroke recurrence. The warfarin dose should maintain a target international normalized ratio (INR) of “2.0–3.0”. (7) Antithrombotic therapy of non-cardiac ischemic stroke and TIA (4 points): The aspirin dose for prevention purpose is “50–325 mg/day”, and the main side effect is “gastrointestinal tract bleeding”. In case of intolerance or allergy to aspirin, clopidogrel can be administered at a dose of “75 mg/day”.

### Statistical analysis

All statistical analyses were completed using SPSS11.5 statistical software. Continuous and normally distributed variables were expressed as mean ± SD, and variables not normally distribution were expressed as median (IQR). Categorical data were described using frequency and percentages. Scores for knowledge about stroke prevention were quantified by taking each knowledge point as 1 point. Each correct answer scored 1 point, and each incorrect or unknown answer scored 0 points. Subscores of pre-hospital stroke recognition and management knowledge and secondary stroke prevention knowledge among community GPs and nurses were calculated. The non-parametric test was performed to compare the effects of different factors on the management capabilities of GPs and nurses for stroke prevention and treatment. Spearman rank correlation analysis was performed on the total score and sociodemographic characteristics about the participants, such as age, gender, and educational level.

## Results

### Questionnaire completion

A total of 331 test questionnaires were returned, for a valid response rate of 69.0%. The participants included 243 community GPs and 88 community nurses.

### General information of participants

Of the 331 GPs and nurses, there were 95 males (28.7%) and 236 females (71.3%), aged 18–68 years, with a mean age of (36.2 ± 10.0) years. The top three specialties of the participants before engaging in community health services were internal medicine (142/331, 42.9%), public health (52/331, 15.7%), and surgery (40/331, 12.1%). A total of 132 (54.3%) GPs and 30 (34.1%) nurses had received GP training. The median time for which all GPs and nurses had engaged in clinical work was 12 years (interquartile range, 5 to 19), and their median time engaged in community health services was 4 years (interquartile range, 1 to 10).

#### Acquisition of stroke prevention and treatment knowledge

Of the GPs and nurses surveyed, 48.0% considered themselves to have or assume the capabilities for managing stroke patients, 39.0% were acquainted with the relevant Chinese guideline for the prevention and treatment of cerebrovascular diseases. Table 1 shows details about the capabilities of the participants for stroke prevention and management. For pre-hospital stroke recognition and treatment, only 7.3% of GPs and nurses correctly provided all five “suddens” of stroke warning signs, whereas 24.5% could not identify any one of them. Only 2.7% GPs and nurses knew pre-hospital stroke screen. For pre-hospital management of acute stroke, only 2.4% of GPs and nurses were completely acquainted with the relevant pre-hospital management knowledge.

**Table 1:** The awareness of community GPs and nurses about ischemic stroke prevention and treatment.

N(%)	GPs (N = 243)	Nurses (N = 88)	Total (N = 331)	P
Guidelines for stroke prevention and treatment	99(40.7)	30(34.1)	129(39.0)	0.273
Classification of strok**e**	161(66.3)	55(62.5)	216(65.3)	0.526
Pre-hospital stroke assessment methods	4(1.6)	5(5.7)	9(2.7)	0.107
Knowledge of stroke warning signs				
Sudden slurred speech, speaking difficulties or understanding difficulties	159(65.4)	47(53.4)	206(62.2)	0.046
Sudden blurred vision of one or both eyes	35(14.4)	16(18.2)	51(15.4)	0.400
Sudden numbness or weakness in one side of the face or limbs	165(67.9)	56(63.6)	221(66.8)	0.467
Sudden severe headache without known cause	83(34.2)	27(30.7)	110(33.2)	0.553
Sudden walking difficulties, dizziness, balance disorders or incoordination	30(12.3)	15(17.0)	45(13.6)	0.270
Management of acute ischemic stroke in community health centers (stations)				
management of airway, breathing and circulation problems	11(4.5)	3(3.4)	14(4.2)	0.891
observation of the heart	15(6.2)	6(6.8)	21(6.3)	0.831
establishment of intravenous access	26(10.7)	10(11.4)	36(10.9)	0.864
inhaled oxygen supply	42(17.3)	14(15.9)	56(16.9)	0.768
assessment of the presence or absence of hypoglycemia	19(7.8)	6(6.8)	25(7.6)	0.761
transfer of patient to a nearby qualified hospital as soon as possible	72(29.6)	23(26.1)	95(28.7)	0.535
Thrombolytic therapy for acute ischemic stroke	126(51.9)	41(46.6)	167(50.5)	0.398
Time window for thrombolytic therapy	48(19.8)	19(21.6)	67(20.2)	0.713
Definition of TIA	119(49.0)	26(29.5)	145(43.8)	0.002
TIA management in community health centers (stations)	48(19.8)	8(9.1)	56(16.9)	0.022
General goal for target BP level for the prevention of recurrent stroke	152(62.6)	55(62.5)	207(62.5)	0.993
Ideal goal for target BP level for prevention of recurrent stroke	117(48.1)	44(50.0)	161(48.6)	0.766
Goal for target HBA1C level for prevention of recurrent stroke	91(37.4)	33(37.5)	124(37.5)	0.993
Goal for BP for prevention of recurrent stroke in hypertensive patients with diabetes	111(45.7)	42(47.7)	153(46.2)	0.741
Awareness of statins therapy for prevention of recurrent stroke	135(55.6)	32(36.4)	167(50.5)	0.002
General goals for LDL-c level for prevention of recurrent stroke	20(8.2)	5(5.7)	25(7.6)	0.438
Goals for LDL-c level for prevention of recurrent stroke in patients associated with multiple risk factors or evidence for atherosclerotic vulnerable plaque or arterial embolism	22(9.1)	9(10.2)	31(9.4)	0.746
Side effects of statins				
abnormal liver function	78(32.1)	12(13.6)	90(27.2)	0.001
muscle damage	49(20.2)	6(6.8)	55(16.6)	0.004
knowing both the side effects	40(16.5)	3(3.4)	43(13.0)	0.002
Awareness of warfarin therapy for prevention of recurrent stroke in patients with atrial fibrillation	94(38.7)	27(30.7)	121(36.6)	0.182
Goal for INR level in patients who receive warfarin therapy	74(30.5)	21(23.9)	95(28.7)	0.242
Dose of aspirin for prevention of recurrent stroke	163(67.1)	49(55.7)	212(64.0)	0.056
The main side effects of aspirin	126(51.9)	26(29.5)	152(45.9)	<0.001
Alternative drugs in patients with intolerance or allergy of aspirin	90(37.2)	19(21.6)	109(33.0)	0.008
Dose of clopidogrel	65(26.9)	15(17.0)	80(24.2)	0.066

TIA, transient ischemic attack; BP, blood pressure; HBA1C, hemoglobin A1C; LDL-c, low-density-lipoprotein cholesterol.

For secondary stroke prevention, only 108 (32.7%) GPs and nurses correctly answered four or more major risk factors, while 97 (29.3%) could not answer any one risk factor. The awareness rates for traditional risk factors were only 64.7% for hypertension, 48.6% for diabetes, and 50.5% for hyperlipidemia. Regarding unhealthy lifestyle including smoking and lack of exercise, the awareness rate was 21.5%. Only 22.7% of GPs and nurses recognized heart disease as a risk factor for stroke. 43.8% of GPs and nurses were acquainted the definition of TIA, but only 14.2% knew about early transfer of TIA patients from community hospital to specialized hospital, there was statistically significant between GPs and nurses (*P <0*.*05*). For drug therapy, the awareness rates for lipid-lowering statins and its side effects were lower in nurses than in GPs (*P <0*.*05*). Meanwhile the awareness rates of the main side effects of aspirin was higher in GPs (*P <0*.*05*).

#### Factors influencing the score of stroke prevention and treatment knowledge

The 331 GPs and nurses obtained a mean subscore of 3.42 ± 2.84 points (median 3 points, interquartile range, 2 to 5) for pre-hospital stroke recognition and management knowledge and a mean subscore of 8.24±5.67 points (median 8 points, interquartile range, 3 to 13) for secondary stroke prevention knowledge. The correct answer rates were 24.4% (3.42/14) and 37.5% (8.24/22), respectively. Regarding the total score of stroke prevention and treatment knowledge, there were significant differences between medical staff with different educational levels, specialties before engaging in community health services, and whether they received GP training (*P* <0.05). Regarding the acquisition of pre-hospital stroke recognition and management knowledge, there were no significant differences between medical staff with different genders, ages, occupation, educational levels, job titles, GP training, clinical experience times and workplace (*P* >0.05). As for the acquisition of secondary stroke prevention knowledge, significant differences were found between medical staff with different occupations, educational levels, specialty before engaging in community health services, and GP training (*P* <0.05).(Table 2).

**Table 2:** Factors influencing the score of stroke prevention and treatment knowledge.

	The subscore for pre-hospital stroke recognition and management knowledge		The subscore for secondary stroke prevention knowledge		The total score for stroke prevention and treatment knowledge	
	Median (IQR)	P	Median (IQR)	P	Median (IQR)	P
Gender		0.180		0.359		0.300
Male	3(2–5)		8(4–13)		11(6–18)	
Female	3(2–4.75)		7(3–12)		10(5–18)	
Age Group (Years)		0.177		0.160		0.390
≤35	3(1–4)		8(4.25–13)		11(6–18)	
36–49	3(2–5)		7(3–13)		11(6–17.75)	
≥50	3(2–4)		7(1–12)		10(4–17)	
Occupation		0.287		0.038		0.090
GPs	3(2–5)		8(4–13)		11(6–18)	
Nurses	3(1–5)		6(2–11)		10(3–17)	
Educational level		0.887		0.006		0.043
Technical secondary school graduates or below	3(2–4)		6(2–11)		9(4–15.5)	
Junior college graduates	3(1–5)		7(3–12)		10(5–17)	
Bachelors	3(2–5)		9(5.75–14.25)		12(7–19)	
Masters or higher degree	3(2–4.5)		9(5.5–19.5)		10(9–23.5)	
Job title		0.391		0.065		0.091
Residents	3(2–4.5)		8(4.5–12)		11(6–18)	
Attending physician	3(2–5)		7(2–13.75)		11(4.24–20)	
Deputy or chief physician	3(0–4)		5(1–8.25)		6.5(3–11.75)	
Specialty before engaging in community health services		0.006		<0.001		<0.001
Internal medicine	3(2–5)		10(5.75–15)		14(7.75–20)	
Surgery	2.5(0–4)		6(2–9)		8(3.25–13)	
Emergency department	1.5(0–3)		6(5–7)		7.5(5–10)	
Others	2(1–4)		7(2–11)		10(4–15)	
GP training		0.119		0.011		0.018
Yes	3(2–5)		9(4–14)		12(6–19)	
No	3(1–4)		7(3–11)		9(5–17)	
Clinical experience time (Years)		0.337		0.622		0.842
0–10	3(1–4)		8(4–13)		10.5(6–18)	
11–20	3(2–5)		9(3–13)		11(4–18)	
21–30	3(2–5)		6.5(1.75–13)		11(4.75–17.25)	
>30	2(2–5.5)		7(1.5–10.75)		9(4.25–17.25)	
Workplace		0.370		0.642		0.803
Community health centers	3(1.5–4.5)		8(4–12)		11(6–18)	
Community health stations	3(2–5)		7(2–14)		10(4–18.25)	

IQR: interquartile range.

The subscores of pre-hospital stroke recognition and management knowledge and secondary stroke prevention knowledge, as well as the total score of stroke prevention and treatment knowledge, were positively correlated with the educational level. That is, the total score of the medical staff’s management knowledge increased with increasing educational level. However, only the correlations with the subscore of secondary stroke prevention knowledge and the total score of stroke prevention and treatment knowledge had statistical significance (*P*<0.05). The subscore of pre-hospital stroke recognition and management knowledge was positively correlated with age, while the subscore of secondary stroke prevention knowledge and the total score of stroke prevention and treatment knowledge were negatively correlated with age(Table 3).

**Table 3:** Factors influencing the knowledge of community GPs and nurses for stroke prevention and treatment.

	Gender		Age		Educational level	
	r_s_	P	r_s_	P	r_s_	P
The subscores of pre-hospital stroke recognition and management knowledge	-0.074	0.180	0.050	0.367	0.043	0.435
The subscore of secondary stroke prevention knowledge	-0.050	0.360	-0.103	0.061	0.192	<0.001
The total score of stroke prevention and treatment knowledge	-0.057	0.300	-0.068	0.218	0.154	0.005

## Discussion

The survey data showed that community GPs and nurses in the major urban districts of Chongqing seriously lack knowledge for either pre-hospital stroke treatment or secondary stroke prevention. For acute ischemic stroke, the use of thrombolytic therapy within 3–4.5 h of onset is presently the only proven effective treatment in the acute stage. The rate of thrombolysis only reached 3.9–10.2% in developed countries [12–15] and in China, a recent report has shown that the rate of intravenous rt-PA thrombolysis for ischemic stroke was as low as 1.3% [16]. The main reason for the low rate of thrombolysis is the pre-hospital delay after stroke onset [14–15, 17–19]. Therefore, community GPs and nurses should perform an early diagnosis and initial management, as well as rapidly transfer stroke patients to qualified hospital as soon as possible to reduce pre-hospital delay. They should master pre-hospital stroke recognition and management related knowledge, including stroke warning signs, pre-hospital stroke assessment method, thrombolytic therapy and its time window, and pre-hospital management of acute stroke patients. In this study, however, the average knowledge score of questionnaire involved pre-hospital stroke recognition and management was only about 1/4 total score, and for each knowledge point, the rate of correct answer was very low. The result showed that community GPs and nurses in the Chongqing urban area urgently need to improve their knowledge about stroke symptom recognition, pre-hospital assessment, and on-site stroke management.

For secondary stroke prevention, the average knowledge score was very low (correct rate 37.5%). Regarding TIA, less than half knew the definition of TIA. Due to the limited auxiliary examination and insufficient management knowledge of medical staff in community health centers (stations), the ideal management of TIA patients is transferring them to a qualified hospital for further examination as soon as possible. The aim is to reduce the risk of stroke attack through management by a neurologist. However, in this study very few participants are acquainted with this knowledge. Regarding stroke risk factors, nearly two thirds of GPs and nurses could recognize hypertension as a risk factor for stroke, whereas awareness rate of the other common risk factors was low, especially only about one fifth of participants recognized unhealthy lifestyle including smoking and lack of exercise as a risk factor for stroke. Given the current high levels of male smoking and the rapid increase in female smoking [20], the community medical staff should improve the awareness of smoking hazards and afterwards strengthen the propaganda of community residents. According to our survey, the participants had not enough knowledge about the control of risk factors, the target level of BP, HbA1C and LDL-C for secondary stroke prevention, the use and side effects of statins. Despite the vast majority were able to correctly guide patients to use aspirin, only one third of the participants correctly identified the preferred anticoagulant for atrial fibrillation. The above results indicated a very low level of knowledge about secondary stroke prevention. Similarly, the survey data from other cities such as Beijing, Liaoning, and Guangxi showed that the level of stroke prevention knowledge among Chinese primary medical staff needs to be improved [21–24]. The recurrence rate of stroke remains high in the Chinese population, due to a lack of risk factor and medication management [25]. Greater effort is required to improve the awareness of stroke risk factors and the control of the stroke risk factors.

Furthermore, in this survey we found that the participants who practiced internal medicine previously and who received GP training had significant higher total score, especially for secondary stroke prevention knowledge, which showed that strengthening GP training would be helpful to improve the acquisition of stroke prevention knowledge. Given that community GPs and nurses play important roles in stroke prevention and pre-hospital treatment in China, further post-graduate education and training to reduce major risk factor and improve medication management is urgently required. In fact, Chinese guidelines for stroke prevention and treatment have made specific recommendations on pre-hospital stroke recognition, treatment and management and secondary stroke prevention. There is a great necessity to strengthen the learning of the clinical guidelines among community GPs and nurses, making the guidelines effectively guide the stroke management in communities. A further recommendation is to redesign the guidelines so they are concise and easy to read and easily accessed by community GPs and nurses.

There are some limitations in this study. Firstly, the survey was conducted in only urban communities in Chongqing, therefore it may not represent non-urban community centers. Secondly, actual practice may not correlate with self-reported knowledge. Thirdly, not all knowledge of stroke management was assessed in the test questionnaires. Despite these limitations a reasonable high response rate of 69% was attained and therefore these results represent current knowledge of GPs and nurses delivering community care to people who have had a stroke in Chongqinq region.

## Conclusion

In summary, the survey results show there is an urgent need for education and training on risk factor management for reducing the incidence of stroke, and on the acute management of stroke among community based GPs and nurses in the Chongqing region in China.
