# Correction: Knowledge of Community General Practitioners and Nurses on Pre-Hospital Stroke Prevention and Treatment in Chongqing, China

**DOI:** 10.1371/journal.pone.0213969

**Published:** 2019-03-13

**Authors:** Juan Yang, Jie Zhang, Shu Ou, Ni Wang, Jian Wang

S1 File is omitted from the list of Supporting Information. It can be viewed below.

## Supporting information

S1 File Self-designed test questionnaire. (DOCX)
