# Supplementary material for: Correction: Knowledge of Community General Practitioners and Nurses on Pre-Hospital Stroke Prevention and Treatment in Chongqing, China
Source: PLoS One. 2019 Mar 13;14(3):e0213969. doi: 10.1371/journal.pone.0213969 (PMC6415895; doi:10.1371/journal.pone.0213969)
Supplement: S1 File — (DOCX) [file pone.0213969.s001.docx]

ID:

**Questionnaire of Knowledge of Community General Practitioners and Nurses on Pre-Hospital Stroke Prevention and Treatment in Chongqing, China**

Note: three types of questions

1. Single choice: please choose the answer most suitable for your according to your current situation, and “**√**” in the box.
2. Gap filling: please fill in the answers you think in the horizontal line, if you don’t do it, using “/” in the line.
3. Answer question: please fill in the answers you think in the blanks of the questions, if you don’t do it, using “/” in the blanks.

**Part One**: general information

1. Gender: □Male □Female
2. Age: _____years
3. Occupation: □Community general practitioners (GPs) □Nurses
4. Educational level: □Technical secondary school graduates or below

□Junior college graduates □Bachelors □Masters or higher degree

1. Job title: □Residents □Attending physician □Deputy physician

□Chief physician

6. Specialty before engaging in community health services: □Internal medicine

□Surgery □Obstetrics and gynecology □Pediatrics □Psychiary □Ophthalmology and otorhinolaryngology □Emergency department □Clinical laboratory and ultrasonic department □Dermatology department □General family medicine □Traditional Chinese medicine □Public health □Others

7. General practitioners training or not: □Yes □No

If yes, training time (Months)______

8. Clinical experience time (Years)______

9. Time since engaging in community health services (Years)_____

10. Workplace: □Community health centers □Community health stations

11. Perceived ability to manage stroke patients in community or not: □Yes □No

**Part Two**: Participants’ knowledge about concepts of stroke, pre-hospital stroke recognition and emergence treatment

12. Know the Chinese guidelines for stroke prevention and treatment: □Yes □No

13. Classification of stroke_________

14. Please list the stroke warning signs:

15. Whether or not to use the pre-hospital stroke assessment methods: □Yes □No

If yes, please list the specific assessment methods

16. How to deal with the acute stroke patients in community?

17. What is the most effective treatment for acute ischemic stroke?

What is the effective time window of this treatment?

**Part Three**: Participants’ knowledge about secondary stroke prevention

18. Please list major risk factors for stroke (at least five factors):

19. The following indicators should be achieved for the drug control of stroke related risk factors

1) For ischemic stroke and TIA, antihypertensive therapy is recommended to reduce the risk of stroke and other vascular events recurrence. Generally, the target of blood pressure (BP) reduction should be less than ___ / ___mmHg, and ideally it should be less than ___ / ___mmHg.

2) For diabetes, the target level of blood glucose control should be glycosylated hemoglobin C (GHbA1C) less than ___ %.

3) For hypertensive patients with diabetes, the goal of BP should be less than ___ / ___mmHg.

4) Ischemic stroke and TIA patients with elevated cholesterol levels are recommended for the use of _____(drug), and the goal is to reduce low-densitylced to _____mmol/L or by ___% among patients associated with multiple risk faipoprotein cholesterol (LDL-C) to ____mmol/L or by ___%; the LDL-C level should be reductors or evidence for intracranial and extracranial aortic atherosclerotic vulnerable plaque or arterialembolism.

5) For ischemic stroke and TIA patients associated with atrial fibrillation, the use of _____(drug) is recommended to prevent stroke recurrence. The dose of the drug should maintain a target international normalized ratio international standardized ratio (INR) of______.

20. The aspirin dose for prevention purpose is ____ mg/day, and the main side effect is_________. In case of intolerance or allergy to aspirin, _____can be administered ,and the dose of the drug is_____mg/day.

21. The main side effects of statins include______ and _________, thus___ and ___ should be monitored regularly. When ___________________, the dosage should be reduced or the drug should be discontinued for observation.
